# Rapid free thiol rebound is a physiological response following cold‐induced vasoconstriction in healthy humans, primary Raynaud and systemic sclerosis

**DOI:** 10.14814/phy2.14017

**Published:** 2019-03-27

**Authors:** Amaal Eman Abdulle, Anniek M. van Roon, Andries J. Smit, Andreas Pasch, Matijs van Meurs, Hendrika Bootsma, Stephan J. L. Bakker, Mohammad Y. Said, Bernadette O. Fernandez, Martin Feelisch, Harry van Goor, Douwe J. Mulder

**Affiliations:** ^1^ Department of Internal Medicine Division Vascular Medicine University of Groningen – University Medical Centre Groningen Groningen The Netherlands; ^2^ Department of Biomedical Research University of Bern Bern Switzerland; ^3^ Department of Critical Care University of Groningen – University Medical Centre Groningen Groningen The Netherlands; ^4^ Department of Rheumatology and Clinical Immunology University of Groningen – University Medical Centre Groningen Groningen The Netherlands; ^5^ Department of Internal Medicine Division of Nephrology University of Groningen – University Medical Centre Groningen Groningen The Netherlands; ^6^ Clinical and Experimental Sciences Faculty of Medicine University of Southampton Southampton United Kingdom; ^7^ Department of Pathology and Medical Biology Section Pathology University of Groningen – University Medical Centre Groningen Groningen The Netherlands

**Keywords:** Free thiols, nitric oxide, Raynaud's phenomenon, redox system, systemic sclerosis

## Abstract

Raynaud's phenomenon (RP) is often the first sign of systemic sclerosis (SSc). Molecular mechanisms involved are incompletely understood, but reactive oxygen, nitrogen, and sulfur species are thought to play an important role in the pathogenesis of SSc. Free thiol groups play a protective role against oxidative stress and may represent an attractive therapeutic target. We aimed to investigate the effects of hypothermia‐induced vasoconstriction on the responsiveness of redox‐related markers. Thirty participants (*n* = 10/group [SSc, primary Raynaud's phenomenon (PRP), healthy controls (HC)]) were included in this study. Fingertip photoelectric plethysmography was performed during a standardized cooling and recovery experiment. Venous blood was collected at four predetermined time points. Free thiols, NO‐derived species (nitros(yl)ated species, nitrite, nitrate), sulfate and endothelin‐1 were measured. Lower baseline concentrations of free thiols were observed in PRP and SSc patients (HC: 5.87 [5.41–5.99] *μ*mol/g; PRP: 5.17 [4.74–5.61]; SSc 5.28 [4.75–5.80], *P* = 0.04). Redox‐related markers remained unchanged during cooling. However, an unexpected increase in systemic free thiol concentrations was observed in all groups during the recovery phase. The response of this marker differed between groups, with a higher increase found in SSc patients (HC Δ = 1.30 [1.48–1.17]; PRP Δ = 1.04 [1.06–1.03]; SSc Δ = 1.72 [1.13–1.49], *P* = 0.04). NO‐derived species, sulfate and endothelin‐1 levels remained unchanged throughout the recovery phase. This exploratory study sheds light on the rapid responsiveness of systemic free thiol concentrations following reperfusion, which may reflect overall redox balance. The robust response to reperfusion in SSc patients suggests that reductive systems involved in this response are functionally intact in these patients.

## Introduction

Systemic sclerosis (SSc) is a complex connective tissue disease of unknown etiology characterized by autoimmunity and severe fibrosis in the skin and internal organs (LeRoy and Medsger 2001; Denton and Khanna 2017). Vasculopathy is the hallmark of this disease and associated with endothelial dysfunction, inflammation, and extracellular matrix remodeling in the vascular wall. Raynaud's phenomenon (RP), which is an episodic vasospastic disorder of the extremities, is the earliest recognizable clinical sign that reflects the vasculopathy in SSc (Herrick 2005). Patients with primary Raynaud's phenomenon, in the absence of an underlying systemic disease, lack these (specific) vascular abnormalities. Increased levels of endothelin‐1 (ET‐1), a potent vasoconstrictor, have previously been reported in both SSc and PRP (primary Raynaud's phenomenon) patients (Zamora et al. 1990; Mostmans et al. 2017). Due to the unavailability of effective therapy, these vascular abnormalities often lead to severe morbidity and premature mortality (Leroy 1988; Herrick et al. 2000; Matucci Cerinic and Kahaleh 2002).

Following Murrell's hypothesis (Murrell 1993), stating that the pathogenesis of SSc is linked to the generation of reactive oxygen species (ROS), several studies have presented evidence that overproduction of ROS – a collective term for oxygen‐derived species with enhanced chemical reactivity – plays an important role in the pathogenesis of SSc (Herrick and Matucci 2001; Sambo et al. 2001; Ogawa et al. 2006). Although chronically elevated levels of ROS have been suspected to prolong disease, oxidative stress (i.e., an imbalance between the production of ROS and antioxidant defences (Jones and Sies 2015)) may also be an initiating factor in the development of SSc vasculopathy. In addition to the well‐known damaging properties of ROS, other reactive species (i.e., reactive nitrogen species [RNS] and reactive sulfur species [RSS]) are also thought to play an important role in SSc (Matucci Cerinic and Kahaleh 2002; Wang et al. 2016). More importantly, the chemical interactions between these reactive species cause homeostatic disruption due to the production of highly reactive free radicals and other secondary metabolites (Nagpure and Bian 2016; Cortese‐Krott et al. 2017). Therefore, these reactive species should be viewed as a unified entity, also referred to as the “reactive species interactome” (Cortese‐Krott et al. 2017). In addition to the increased production of free radicals, some have shown that patients with SSc also exhibit a decreased level of antioxidants, and enzyme activities related to the defence against oxidative stress (Herrick and Matucci 2001; Ogawa et al. 2010). More particularly, endothelial cells (ECs) were previously found to be catalase deficient and, therefore, are more prone to ROS injury (Jornot and Junod 1992). This deficiency may eventually initiate a self‐perpetuating cycle of recurrent RP attacks, increased ROS production, ischemia/reperfusion (I/R) injury, and an inability to defend against further stress.

Free thiol (i.e., sulfhydryl groups) are thought to play a protective role against oxidative stress through ROS scavenging, and thereby function as sentinels of distant danger (Cortese‐Krott et al. 2017). In addition, free thiols are also believed to be part of an intricate network of the redox system which regulates crucial roles in proteins, and may lead to adjustment of protein structures and enzymatic functions according to the redox state (Go and Jones 2011). Free thiols can be oxidized by ROS and other reactive species, and are active components of antioxidant buffer capacity, implicating that their extracellular level may be a direct reflection of overall redox status (Banne et al. 2003; Chung et al. 2013; Turell et al. 2013; Cortese‐Krott et al. 2017). Therefore, chronic systemic oxidative stress can simply be measured as the depletion of the free thiols in serum, which mainly consists of the single free SH‐group of human serum albumin (Banne et al. 2003). Several studies have previously demonstrated clear evidence that free thiols are an indirect measure of systemic oxidative stress. For instance, in I/R related disorders such as myocardial stunning, researcher have previously shown a loss of sulfhydryl groups following I/R. Moreover, in other conditions (e.g., chronic heart failure and renal transplantation) thiol depletion predicted mortality and disease outcome and was associated with cardiovascular disease (Koning et al. 2015, 2016).

A better understanding of the role played by oxidative stress during a Raynaud's attack could help to identify early interventions aimed at delaying the onset of overt symptoms and/or attenuating the course of the disease. The aim of this exploratory study was threefold. First, we sought to investigate whether redox‐related makers (free thiols, NO‐derived species and sulfate [stable end product of hydrogen sulfide]) measured at baseline differ between groups. Second, we wanted to assess the difference in effects of hypothermia induced vasoconstriction on the responsiveness, as defined as the change (delta) during cooling and recovery, of redox related markers between SSc patients, PRP patients and healthy controls (HC). Lastly, we aimed to investigate whether there is an association between the deltas of these markers. We hypothesized that a lower steady‐state concentration of free thiols would be observed in patients with SSc, due to the chronic inflammation and impaired antioxidative defence mechanism. In addition, we anticipated to find a difference in response to recovery in comparison to the initial vasospastic event, due to the assumption that reperfusion can be potentially more harmful than ischemia itself.

## Materials and Methods

### Ethical approval

This study was conducted in accordance with the Declaration of Helsinki. Study approval was obtained from the local Medical Ethics Committee of the University Medical Centre Groningen (METc 2015/219), the Netherlands, and all participants provided written informed consent. This study was registered in the research database of the UMCG (NL53132.042.15).

### Patient population

This exploratory study was conducted between 2015 and 2016 at the University Medical Centre Groningen (the Netherlands). Three groups of participants all aged ≥18 years were included, group 1 comprised of healthy controls without a history of RP (*N* = 10), group 2 included patients with PRP (*N* = 10), and group 3 composed of patients with RP secondary to SSc (*N* = 10). All SSc patients fulfilled the American College of Rheumatology/European League Against Rheumatism (ACR/EULAR) 2013 criteria (Van Den Hoogen et al. 2013). PRP patients had negative serology, normal nailfold capillaries assessed with capillaroscopy and no clinical history of digital ulcers. Diagnosis of PRP was confirmed by the treating physician. Subjects were excluded in case of inaccessibility of the photoelectric plethysmograhy (PPG) measurements.

### Photoelectric plethysmography of the fingers during cooling and recovery

**Figure 1:**
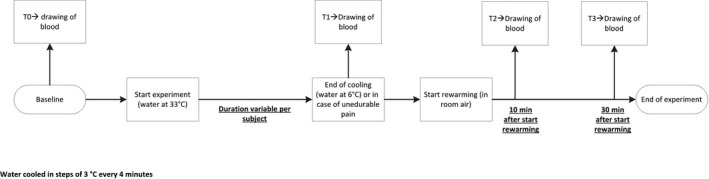
Schematic overview of the study experiment. “Cooling” refers to a temperature ramp between 33°C and 6°C implemented over 36 min.

A standardized cooling and recovery experiment was performed (Fig. 1) and concomitantly PPG was recorded as described previously (Wouda 1977; Suichies et al. 1992; van Roon et al. 2016). All subjects were asked to refrain from alcohol intake and smoking at least 8 h before the assessments. Before the measurements, patients were allowed to acclimatize for half an hour in a room with a stable temperature of 23–24°C. During the experiment, the left hand of the subject (to the level of the radiocarpal joint) was submerged in a water bath of 33°C with PPG sensors attached to each finger, enabling simultaneous measurement of perfusion in all five digits. Water was cooled every 4 min by 3°C, for 36 min in total, with water at 6°C at the end of cooling. During each step, there was a stabilization period of 4 min for each temperature, after which 15 sec of perfusion was recorded. In case of unendurable pain the cooling experiment was discontinued and that time was marked as time point 1 (T1). After the cooling steps, the hand was taken out of the water, padded dry and then rested on a dry towel to track recovery of perfusion in the open air for 10 min (time point 2). Perfusion was recorded during the last 15 sec of every minute during recovery. Mean ischemic time (minutes) was calculated for all fingers and was defined as the mean time of perfusion loss during cooling and recovery. PPG pulses were analyzed from R‐peak (detected in the ECG) up to 600 msec. The mean (SD) of the amplitude of the pulses was determined, and a signal‐to‐noise ratio (*S*/*N*) was calculated. Perfusion was defined as *S*/*N* > 15 (van Roon et al. 2016). The experiment was deemed positive for RP when ≥2 fingers lost perfusion and stayed abnormal during ≥2 consecutive steps. Data acquisition was performed using a Biopac MP‐100 system with five PPG100C amplifiers and PPG200C sensors (infrared light, 860 nm), an ECG100C amplifier with ECG‐cables, an SKT100C amplifier and TSD202C temperature sensor, and AcqKnowledge 3.8.2 software (Biopac Systems Inc., Goleta, CA). All signals (ECG, temperature and 5 PPG's) are sampled at 100 Hz and stored in a file for off‐line processing with dedicated software to determine pulsations in each finger.

### Clinical and laboratory assessments

The following patient characteristics were obtained: sex, age, smoking behavior, comorbidities, body weight (kg), length (cm), and use of medication. Furthermore, the presence of digital ulcers (DU) was documented and clinical evaluations including serum autoantibodies profile, esophageal scintigraphy and pulmonary function tests were performed in all SSc patients.

Blood was drawn from the antecubital vein of the left arm at four time points: at baseline (T0), at the end of the cooling experiment (T1), and 10 min (T2) and 30 min (T3) after start of recovery. Plasma and serum samples were stored at −80°C and thawed just before use. Prior to storing, samples were centrifuged at 1300 ***g*** at room temperature for 10 min. Markers of oxidative stress (free thiols), NO‐derived species and sulfate were measured. Nitros(yl)ated species (RXNO), nitrite, nitrate, and free thiols (corrected for proteins) were analyzed by gas‐phase chemiluminescence and ion chromatography as previously described (Feelisch et al. 2002; Rassaf et al. 2002; Koning et al. 2016). Serum levels of ET‐1 were measured according to the manufacturer's instructions using commercially available Quantikine ELISA kits (DET10 R&D Systems, Abingdon, UK) as previously described (Jongman et al. 2014). Plasma sulfate was measured by ion chromatography, as described earlier (Koning et al. 2017).

### Statistical analysis

Statistics were performed with The Statistical Product and Service Solutions (SPSS; version 22, Released 2013, IBM Corp., Armonk, NY). The data of this pilot study are presented as mean ± standard deviation for normally‐distributed data and median (interquartile range) for data that was nonnormally distributed. Given the design of the study, the experiment was analyzed in two parts: from time point 0 to time point 1 (cooling) and from time point 1 to time point 3 (recovery). Differences between groups at baseline were analyzed using nonparametric test (Kruskal–Wallis_,_ Mann–Whitney U). The main objective (difference in responsiveness of the markers between groups during cooling and recovery as measured by the delta) was assessed using paired nonparametric test (Wilcoxon signed‐rank test). In addition, correlations between the calculated deltas of the markers during cooling and mean ischemic time during cooling were determined by either Pearson or Spearman correlation coefficients. In addition, we also assessed the association between the calculated deltas of the markers during recovery and mean ischemic time during recovery. A *P*‐value of ≤0.05 was regarded as statistically significant.

## Results

### Patient characteristics

In total, 32 subjects were found eligible to participate in the study. Two subjects were excluded due to an inaccessible PPG measurement, thus 30 subjects were included in the analysis. Characteristics of the study participants are displayed in Table 1. The median age of all subjects was 43 years (range 18–73) and 20 (HC *n* = 7, PRP *n* = 8, SSc *n* = 5) of them were females. Raynaud's attack was successfully induced in all PRP and SSc patients, but not in the healthy controls. Three PRP patients and 7 SSc patients prematurely ended the cooling part of the experiment due to unendurable pain. Time at which unendurable pain had occurred was marked as time point 1 (end of cooling).

**Table 1:** Patient characteristics of healthy controls, patients with primary Raynaud's phenomenon and systemic sclerosis

	Healthy controls *N* = 10	Primary Raynaud phenomenon *N* = 10	Systemic sclerosis *N* = 10
Age (years), mean ± SD	29.9 ± 2.7	47.8 ± 16.2	56.2 ± 11.5
Female gender, *n*	7	8	5
Weight (kg), mean ± SD	71.3 ± 11.7	64.4 ± 11.9	73.3 ± 11.3
Height (cm), mean ± SD	177 ± 8.7	170 ± 10.0	176 ± 5.9
Smoking, *n*			
Current	0	1	2
Past smoker	0	3	7
Comorbidities, *n*			
Hypertension	0	1	2
Hypercholesterolemia	0	0	1
Diabetes	0	1	0
ACR/EULAR score ≥9, *n*	0	0	10
Disease duration (years), median (IQR)	N/A	8.0 (4.0‐17.0)	4.5 (3.0‐6.3)
Abnormal NCM pattern, *n*	0	0	10
Positive autoantibodies, *n*	0	0	8
Organ involvement, *n*			
Pulmonal	0	0	0
Esophageal	0	0	3
Creatinine, median (IQR)	72.1 [66.5‐77.6]	72.0 [63.5‐72.0]	76.2 [63.5‐88.6]
eGFR, median (IQR)	94.5 [90.6‐114.8]	79.3 [68.3‐97.4]	86.6 [74.1‐98.5]
Digital ulcers, *n*	0	0	6
Medication, *n*			
Nifedipine	0	1	2
Iloprost	0	0	2
Bosentan	0	0	3

ACR/EULAR, The American College of Rheumatology/European League Against Rheumatism (EULAR); NCM, Nailfold Capillary Microscopy.

### Digital perfusion during standardized cooling and recovery experiment

**Figure 2:**
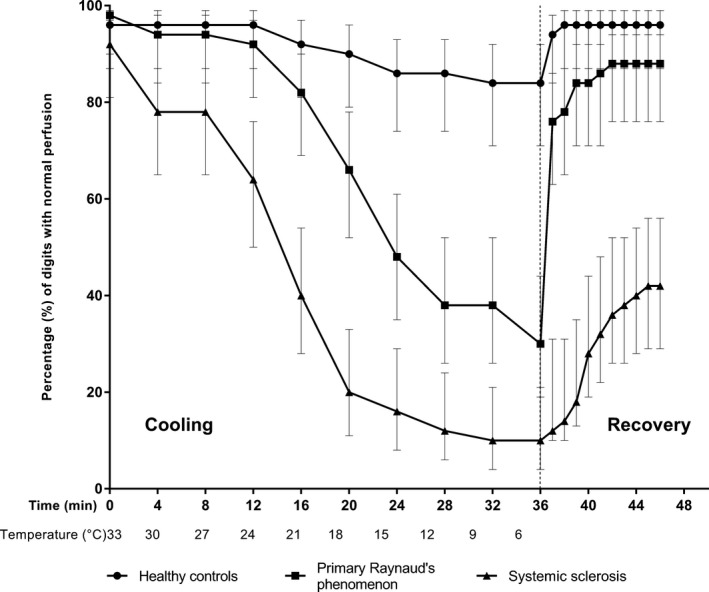
Digital perfusion during cooling and recovery experiment. Percentage of digits with normal perfusion (95% CI) assessed with photoelectric plethysmography during stepwise cooling and recovery. Lines represent healthy controls (*n* = 10), patients with primary Raynaud (*n* = 10) and SSc patients (*n* = 10). The vertical dashed line indicates the beginning of the recovery phase. All healthy controls completed the entire cooling duration of 36 min. Seven primary Raynaud patients completed the cooling experiment, two patients discontinued after 32 min (9°C), and one patient discontinued after 12 min (24°C). In addition, three systemic sclerosis patients completed the cooling experiment, one patient discontinued after 20 min (18°C), one patients discontinued after 24 min (15°C), two patients discontinued after 28 min (12°C), and three patients discontinued after 32 min (9°C). In addition to the cooling phase, perfusion of all participants was measured during the recovery phase. HC, healthy controls; PRP, primary Raynaud's phenomenon; SSc, systemic sclerosis.

The percentage of digits with normal perfusion assessed with PPG during the cooling and recovery experiment is depicted in Figure 2. A longer duration of mean ischemic time was observed in SSc patients (30 min, 24–37 min) compared to PRP (12 min, 9–14 min, *P* = 0.01) and HC (1,1 min, 0–3.7 min, *P* < 0.001). Also, a considerably longer recovery time was found in the SSc group compared to the PRP group (SSc: 8 min [4–10 min]; PRP: 1.1 min [1.0–2.1 min], *P* < 0.001), and about 50% of the baseline perfusion recovered 10 min after cessation of cooling stress in SSc patients.

### Baseline comparison of circulating biomarkers

**Figure 3:**
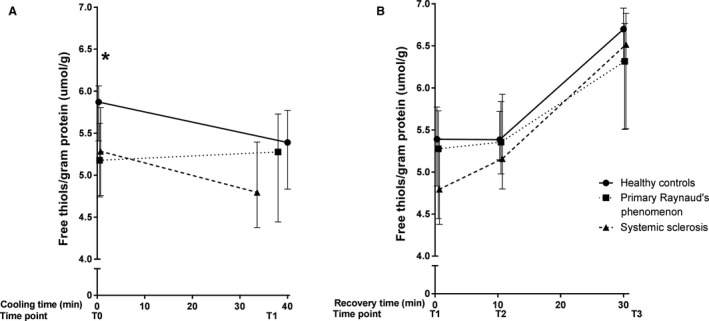
Median (IQR) concentration of free thiols during cooling (A) and recovery (B). Measurements at four different time points during a standardized cooling and recovery experiment in healthy controls (*n* = 10 at all time points), primary Raynaud (*n* = 10 during cooling, *n* = 9·10 min after start recovery and *n* = 8 at the end of recovery) and systemic sclerosis patients (*n* = 10 during cooling and 10 min after start recovery, *n* = 9 at the end of recovery). The asterisk represents a significant difference between groups (*P* < 0.05). During cooling: HC Δ = −0.48 [−0.57 to −0.29], PRPΔ = 0.09 [−0.29–0.11], SSc Δ = −0.48 [−0.38 to −0.41]. During recovery: HC Δ = 1.30 [1.48–1.17]; PRP Δ = 1.04 [1.06–1.03]; SSc Δ = 1.72 [1.13–1.49].

**Figure 4:**
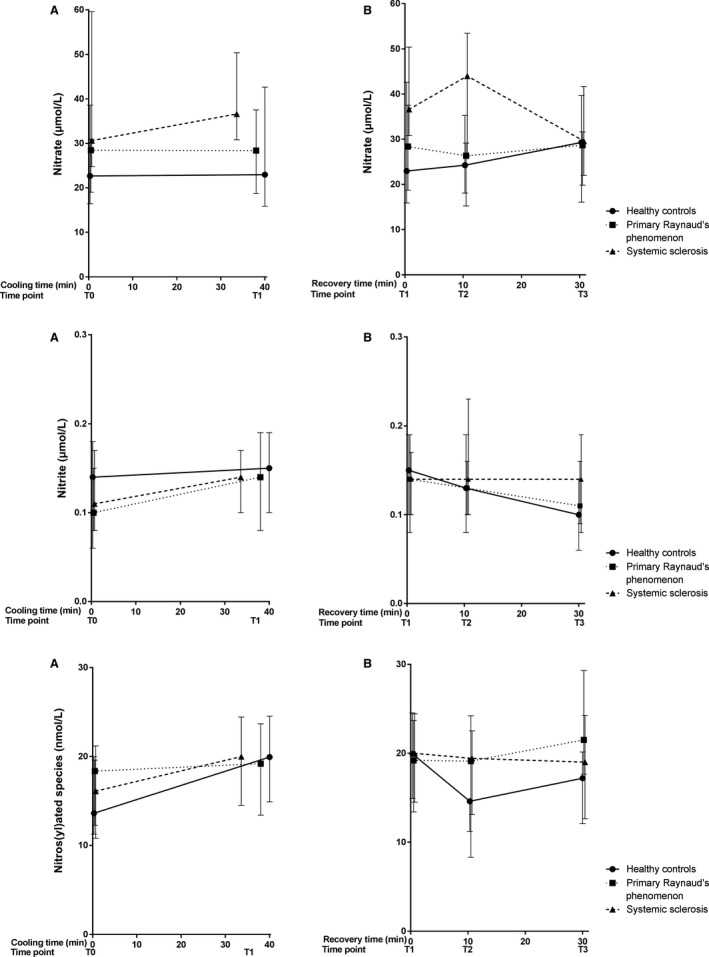
Median (IQR) concentration of NO‐derived species (nitrite, nitrate and RXNO) during cooling (A) and recovery (B). Measurements at four different time points during a standardized cooling and recovery experiment in healthy controls (*n* = 10 at all time points), primary Raynaud patients (*n* = 10 during cooling, *n* = 9 10 min after start recovery and *n* = 8 at the end of recovery) and systemic sclerosis patients (*n* = 10 during cooling and 10 min after start recovery, *n* = 9 at the end of recovery). During cooling: HC: nitrate: Δ = 0.28 [−0.53–4.04], nitrite: Δ = 0.01 [0.03–0.008], RXNO: Δ = 6.32 [3.65–4.56]; PRP: nitrate: Δ = −0.09 [−0.31–6.57], nitrite: Δ = 0.03 [0.0004–0.04], RXNO: Δ = 0.84 [1.16–4.09]; SSc: nitrate: Δ = 5.65 [6.03 to −9.17], nitrite: Δ = 0.03 [0.01–0.004], RXNO: Δ = 3.88 [3.72–3.24]. During recovery: HC: nitrate Δ = 6.34 [0.18 to −2.99], nitrite Δ = −0.05 [−0.03 to −0.04], RXNO Δ = −2.74 [−2.80 to −4.38]; PRP: nitrate Δ = 0.29 [1.07 to −5.89], nitrite Δ = −0.03 [0.001 to −0.03], RXNO Δ = 2.30 [4.22–5.61]; SSc nitrate Δ = −7.01 [−8.83 to −8.71], nitrite Δ = −0.007 [−0.02–0.02], RXNO Δ = −0.98 [−1.88 to −0.18].

**Figure 5:**
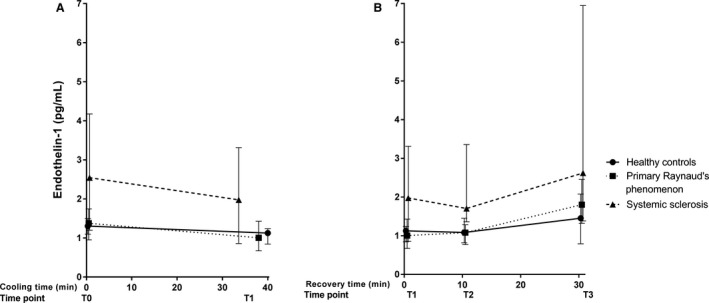
Median (and IQR) concentration of endothelin‐1 during cooling (A) and recovery (B). Measurements at four different time points during a standardized cooling and recovery experiment in healthy controls (*n* = 10 at all time points), primary Raynaud (*n* = 10 during cooling, *n* = 9·10 min after start recovery and *n* = 8 at the end of recovery) and systemic sclerosis patients (*n* = 10 during cooling and 10 min after start recovery, *n* = 9 at the end of recovery). During cooling: HC: Δ = −0.18 [−0.25 to −0.26]; PRP: Δ = −0.37 [−0.28 to −0.31]; SSc: Δ = −0.56 [−0.34 to −0.86]. During recovery: HC Δ = 0.33 [−0.05–0.84]; PRP Δ = 0.79 [0.64–1.02]; SSc Δ = 0.64 [0.52–3.63].

**Figure 6:**
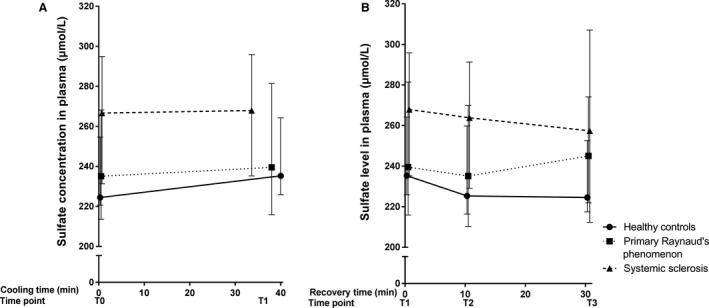
Median (and IQR) concentration of sulfate during cooling (A) and recovery (B). Measurements at four different time points during a standardized cooling and recovery experiment in healthy controls (*n* = 10 at all time points), primary Raynaud (*n* = 10 during cooling, *n* = 9·10 min after start recovery and *n* = 8 at the end of recovery) and systemic sclerosis patients (*n* = 10 during cooling and 10 min after start recovery, *n* = 9 at the end of recovery). During cooling: HC: Δ = 10.80 [5.25–9.55]; PRP: Δ = 4.45 [2.35–13.28]; SSc: Δ = 1.25 [3.93–0.95]. During recovery: HC Δ = −10.70 [−8.42 to −11.65]; PRP Δ = 5.55 [6.09 to −7.29]; SSc Δ = −10.50 [−22.95–11.25].

Baseline median levels of free thiols for the groups are illustrated in Figure 3. Significantly lower concentrations of total free thiols, indicating higher oxidative stress, were observed in PRP and SSc patients, compared to healthy controls (HC: 5.87 [5.41–5.99] *μ*mol/g; PRP: 5.17 [4.74–5.61] *μ*mol/g protein; SSc 5.28 [4.75–5.80] *μ*mol/g protein, *P* = 0.04). No significant differences between PRP and SSc were observed (*P* = 0.59). Although nitrate, sulfate and ET‐1 tended to be higher in SSc patients, no significant differences between groups were observed in baseline levels of NO‐derived species (nitrate: *P* = 0.23; nitrite: *P* = 0.88; RXNO: *P* = 0.88, Fig. 4), ET‐1 (*P* = 0.24, Fig. 5) and sulfate (*P* = 0.14, Fig. 6).

### Differences in responsiveness of the markers between groups during cooling (time point 0 to time point 1) and during recovery (time point 1 to time point 3)

During cooling, free thiols, NO‐derived species, sulfate, and ET‐1 remained unchanged. As shown in Figures 3, 4, 5, the deltas of free thiols, NO‐derived species, and ET‐1 were comparable between groups (free thiols *P* = 0.27; nitrate *P* = 0.64; nitrite *P* = 0.99; RXNO *P* = 0.48, ET‐1 *P* = 0.77). In contrast, circulating sulfate concentrations were significantly different between groups, and the highest delta was observed in the healthy controls (*P* = 0.03, Fig. 6).

During the recovery phase, we found that free thiol concentrations not only recover during reperfusion, but reach levels that exceed baseline concentrations. This finding was coincided with stable concentrations of the gasotransmitter end‐products nitrite and nitrate (in part, originating from NO) and sulfate (related to H_2_S). Moreover, the delta of free thiol concentrations was significantly different between groups (Fig. 3, *P* = 0.04). However, those of the NO‐derived species, ET‐1, and sulfate were comparable between groups (nitrate *P* = 0.68; nitrite *P* = 0.89; RXNO *P* = 0.06; sulfate *P* = 0.31; ET‐1 *P* = 0.25).

### Correlations between laboratory markers and mean ischemic time during cooling and recovery

We investigated the correlation between the delta of these markers and mean ischemic time during cooling and recovery, but found no significant correlations. Furthermore, correlations between the markers were assessed during cooling and recovery. During cooling, we observed a positive correlation between delta nitrate and nitrite (*r* = 0.54, *P* < 0.01), delta sulfate and nitrite (*r* = 0.39, *P* = 0.03) and delta sulfate and free thiols (*r* = 0.48, *P* < 0.01). During recovery, only the positive correlation between delta nitrate and nitrite was found to be significant (*r* = 0.44, *P* = 0.02).

## Discussion

This pilot study demonstrated that circulating free thiol concentrations are lower at baseline in PRP and SSc patients compared to HC. Moreover, we observed a rapid increase in free thiols in all groups during recovery following transient hypothermia. Our findings coincided with stable concentrations of the gasotransmitter end‐products nitrite and nitrate (in part, originating from NO) and sulfate (related to H_2_S). These results not only suggest that RP patients (i.e., primary and secondary) have chronically increased levels of oxidative stress, but also indicate that free thiol concentrations are rapidly elevated following transient hypothermia‐induced vasoconstriction. The latter was an unexpected finding that warrants further investigation.

Raynaud's phenomenon in SSc patients often precedes the onset of other symptoms (e.g., organ involvement) by several years. Although primary and secondary RP share some similarities from a pathophysiological point of view, secondary RP is more likely to be associated with severe complications of vascular insufficiency due to endothelial abnormalities (Houtman et al. 1985; Herrick 2005, 2012, 2017; Flavahan 2015). In support, the potent vasoconstrictor ET‐1 was previously suggested to play an important role in the pathogenesis of SSc vasculopathy (Silveri et al. 2001; Avouac et al. 2013; Mostmans et al. 2017). Although we did observe higher concentration of ET‐1 in SSc patients, this marker remained stably elevated throughout the experiment. This finding is in accordance with a previous study conducted by Smyth and colleagues (2000) who did not find clear differences during a cooling experiment. Collectively, these findings suggest that ET‐1 is not involved in changes in vascular tone during transient hypothermia.

This study showed a significantly lower baseline level of free thiols in both PRP and SSc patients as compared to HC, which may be indicative of chronically increased levels of oxidative stress. Oxidative stress arises when the production of ROS outweighs the buffering capacity of antioxidant defences, eventually leading to cellular damage (Jones and Sies 2015). Following oxidation, serum thiols are not easily reduced compared to their intracellular counterparts, indicating that they are a relatively stable reflection of the systemic redox status. Consistent with this assumption, a marked reduction in serum protein thiols was previously observed in active SSc (Lau et al. 1992; Banne et al. 2003; Giovannetti et al. 2012). This difference could also be explained by the difference in smoking behavior between the groups. For instance, a current hypothesis is that the damaging properties of smoking can even occur after cessation of smoking. Therefore, SSc patients may experience prolonged oxidative‐stress induced damage even after cessation of smoking.

Interestingly, our analyses revealed an additional unexpected biomarker feature, that is, a significant increase in concentration of free thiols during the recovery phase in all groups. Specifically, we here demonstrate that free thiol concentrations not only recover during reperfusion, but reach levels that exceed baseline concentrations. This increase in free thiols (we here define as “thiol rebound”) could be explained by several mechanisms. First, our findings may be the result of rapid reversibility and dynamic nature of processes related to oxidative thiol modification, which lowers the availability of total free thiols (reduced thiols) in the circulation. In a previously conducted experimental study in mice, the protective mechanism in endothelial function following *I*/*R* of thioredoxin, a disulfide‐reducing protein, was shown (Subramani et al. 2016). A similar mechanism could also play a role in hypothermia induced Raynaud's attack. Secondly, this rapid responsiveness of free thiols may be the result of a physiological acute response to reperfusion, which may alter the oxidative balance. This hypothesis is supported by the fact that this thiol rebound phenomenon was also observed in healthy controls. Third, as overall thiol redox status is presumably affected by NO‐derived species (Cortese‐Krott et al. 2017), it is conceivable that NO plays an important role in the processes related to thiol modification, that could have led to the previously mentioned thiol rebound. However, we have to address the fact that no significant increase in nitrate, or any other NO‐derivative measured was observed in healthy controls and PRP patients, despite the presence of the thiol rebound during reperfusion. This observation may indicate that free thiols can be produced/modified independently of NO‐derived species.

Hydrogen sulfide (H_2_S), which is one of the three main gasotransmitters, has been reported to have vasodilator and antioxidant capacity. Therefore, a current hypothesis is that H_2_S may play a more prominent role in the development SSc vasculopathy. In this study we investigated the effect of hypothermia on plasma concentrations of sulfate, which is a stable end product of H_2_S. We observed a trend in elevated plasma levels of sulfate in SSc patients throughout the entire experiment. Urinary sulfur metabolites, such as sulfate, were previously found to be associated with renal function, favorable cardiovascular risk profile and an improved survival in renal transplant recipients (van den Berg et al. 2014) However, as none of the participants had an impaired kidney function our findings are unlikely explained by disturbances in kidney function. In addition, as we also observed a trend towards increased levels of both nitrate and sulfate in SSc patients, it is plausible that these markers of the redox system interact with one another through the same pathway; underlining the complexity of the redox interactome. The fact that both chemical compounds are known dietary constituents complicates the interpretation of these results inasmuch as we cannot exclude that differences in the consumption of nitrate and sulfate containing nutrients may have contributed to the observed changes. Clarification of the interaction of these signaling molecules in the pathogenesis of PRP and SSc awaits further studies.

The exploratory study has some limitations. First, it could be argued that that the aging process (i.e., older age) itself potentially influences the level of oxidative stress. For instance, several studies have shown that ageing cells accumulate increased levels of oxidant‐damaged DNA, which may further increase the level of oxidative stress. Therefore, the large age‐range could potentially affect the baseline level of free thiols. Despite this difference in age between the groups, we have demonstrated that all three groups show the “thiol rebound” phenomenon. This finding may suggest that the response of free thiol level following hypothermia induced vasoconstriction, is not influenced by age. However, in order to investigate the effect of age on the redox status large age‐matched control‐group has to be included. Second, we acknowledge the fact that the differences in gender between groups may play an important role in the oxidative balance. Given the small number of males included in this study, we were unable to demonstrate gender differences. However, this information could give us valuable insight that could potentially clarify the complexity of the redox system, and, therefore, future studies should investigate these gender differences. Third, other factors such as blood pressure, cholesterol and co‐morbidities were not assessed in this study. However, given the fact that the majority of the participants, did not show signs of hypertension or hypercholesterolemia 1 year prior and/or 1 year after the experiment (as assessed by the caregiving rheumatologist or internist), it would be unlikely that the inclusion of these factors would influence our result. Fourth, patients were not asked to discontinue their medication. As many of the vasoactive medication may alter the oxidative balance, it is possible that that the “thiol rebound” phenomenon as observed in SSc patients could potentially be even higher. Fifth, due to the small sample size it was not feasible to analyze differences between SSc patients with reperfusion and those with prolonged ischemia. However, based on the results of this study it is possible to calculate effect sizes, and this will assist future studies in their sample size calculation. Sixth, some patients did not complete the cooling experiment because of unendurable pain and objective documentation of the development of ischemia was lacking in these patients. However, the majority of these patients were SSc patients, and no one in the healthy control group prematurely discontinued the experiment. It is therefore conceivable that these SSc patients prematurely discontinued the experiment due to an RP attack. Despite these limitations, we were able to demonstrate clear differences in the levels of circulating free thiols during the course of a unique cooling and recovery experiment.

In conclusion, this study demonstrates decreased circulating free thiol concentrations in primary RP and SSc patients as compared to healthy controls. Moreover, an unexpected rapid increase in free thiols was present in all individuals during the reperfusion phase of the standardized cooling experiment. The rapid responsiveness of this redox related marker demonstrates the dynamic nature of this seemingly simple biomarker and may be the result of a physiological acute response to reperfusion, which may alter the oxidative balance. The observed response to reperfusion in SSc patients suggests that systems involved in the thiol responsiveness are functionally intact in these patients. Whether a further enhancement of this response, through therapeutic intervention, confers additional protection in SSc and RP remains to be investigated.

## Conflict of Interest

The authors declared no conflicts of interest.
